# The piRNA pathway responds to environmental signals to establish intergenerational adaptation to stress

**DOI:** 10.1186/s12915-018-0571-y

**Published:** 2018-09-18

**Authors:** Tony Belicard, Pree Jareosettasin, Peter Sarkies

**Affiliations:** 1 MRC London Institute of Medical Sciences, Du Cane Road, London, W12 0NN UK; 2 Institute of Clinical Sciences, Imperial College London, Hammersmith Hospital Campus, Du Cane Road, London, W12 0NN UK

## Abstract

**Background:**

piRNAs have a constitutive role in genome defence by silencing transposable elements in the germline. In the nematode *Caenorhabditis elegans*, piRNAs also induce epigenetic silencing of transgenes, which can be maintained for many generations in the absence of the piRNA pathway. The role of multi-generational epigenetic inheritance in adaptation to the environment is unknown.

**Results:**

Here, we show that piRNA biogenesis is downregulated in response to a small increase in temperature. Some effects on gene expression persist into subsequent generations and are associated with a negative fitness cost. We show that simultaneous infection with pathogenic bacteria suppresses downregulation of the piRNA pathway in response to increased temperature. This effect is associated with increased fitness of progeny of infected animals in subsequent generations.

**Conclusions:**

Our results show that the piRNA pathway integrates inputs from the environment to establish intergenerational responses to environmental conditions, with important consequences for the fitness of the subsequent generation.

**Electronic supplementary material:**

The online version of this article (10.1186/s12915-018-0571-y) contains supplementary material, which is available to authorized users.

## Background

In animals, the physical separation of germline and soma, established early in development, is believed to result in the Weismann barrier whereby hereditary information moves only from genes to body cells. However, this barrier is not absolute: a growing number of studies have shown that information may be transferred from soma to germline, triggering epigenetic responses that last for multiple generations. By convention, these phenomena are defined as transgenerational when the response is observed after two or more generations (F2 individuals) and responses observed in the first generation (F1) are known as intergenerational [1]. Often, these effects result from exposing animals to stressful conditions. For instance, bacterial infection of *Tribolium castanatum* can increase resistance of progeny [2], and in mice, severe maternal undernutrition can result in the development of insulin resistance in subsequent generations [3]. The nematode *Caenorhabditis elegans* has been developed as a model to explore molecular mechanisms of multi-generational epigenetic responses to stress. Experiments using starvation [4] or heat stress [5, 6] demonstrated that environmental alterations result in long-term perturbation of gene expression corresponding to the accumulation of small silencing RNAs targeted to specific genes, which persist in the absence of the environmental trigger. However, how environmental perturbations signal to small RNA pathways, including how the germline-soma barrier is crossed, is still unknown.

Any molecular mechanism that accounts for epigenetic phenomena triggered by stress must fulfil two properties: it must be active in the germline thus able to transmit its effects into the next generation, and its activity must be modulated in response to specific signals from the soma. These considerations suggest that the Piwi-interacting small RNA pathway may be a candidate for transmitting epigenetic information. Piwi-interacting small RNAs are 21–33 nt RNAs produced from single-stranded precursor RNAs, which interact with the Piwi family of Argonaut proteins to bring about target recognition and silencing [7]. piRNAs are widely conserved across eukaryotic organisms and are characteristically enriched in the germline where they are often involved in silencing transposable element (TE) expression [7]. In most organisms studied so far, piRNAs are essential for fertility, either directly linked to their TE suppression function or through other, less well-understood roles in germline stem cell maintenance [8].

The functions attributed to the piRNA pathway means that it has typically been considered as constitutive, essential to maintain genome integrity against TE-derived mischief. Nevertheless, the silencing triggered by piRNAs establishes transgenerational effects [9–12]; thus, mechanistically they have the potential to act in transmission of signals from soma through the germline into the next generation. However, it is unknown whether silencing triggered by the piRNA pathway can respond dynamically to the environment; thus, the relevance of piRNAs to stress-induced multi-generational epigenetic phenomena is still unclear. Here, we use the model nematode *C. elegans* to explore environmental regulation of piRNAs.

In *C. elegans*, the bulk of piRNAs are produced from individual loci in two clusters on Chromosome IV, each demarcated by a GTTTC consensus motif [13–15]. These motif-dependent piRNAs are dependent on the RNA pol III factor *gei-11/snpc-4* [16] and the dedicated piRNA biogenesis gene *prde-1* [17]. In addition, a smaller number of motif-independent piRNAs are found scattered through the genome, which do not depend on *prde-1* for their function and for which specific biogenesis factors are still unknown [17, 18]. Despite distinct biogenesis, both types of piRNAs bind to the piwi protein *prg-1* [18] and induce target silencing through recruitment of RNA-dependent RNA polymerases for 22G-RNA production [17]. Motif-dependent and motif-independent piRNAs target different sets of genes: whilst motif-dependent piRNAs target transposable elements and poorly conserved genes with few clear functions, motif-independent piRNA targets are strongly enriched for innate immune genes [17].

Transgenes subjected to piRNA-induced silencing demonstrated that once established, the silent state is maintained for multiple generations in the absence of piRNAs [9–11, 19]. Some endogenous genes targeted by piRNAs are also subject to transgenerational silencing maintained in the absence of piRNAs [20, 21]. Although the mechanism underlying this process is largely understood, what is missing is a coherent account of whether the process is responsive to changes in environmental conditions and thus whether the piRNA pathway might account for transgenerational epigenetic effects important for the natural lifecycle of *C. elegans*.

Here, by investigating the strength of piRNA-dependent silencing in response to different temperatures, we find that motif-dependent piRNA biogenesis is reduced by a moderate increase in temperature from 20 to 25 °C. A subset of gene expression changes induced by this shift persists into subsequent generations after returning to 20 °C. Additionally, we find that the fitness of the progeny of animals grown at 25 °C is reduced relative to the progeny of animals grown at 20 °C. Intriguingly, infection with pathogenic bacteria suppresses the reduction in motif-dependent piRNAs and increases the fitness of progeny relative to the progeny of uninfected animals. Together, our data suggest that piRNAs respond dynamically to environmental stimuli, modifying gene expression and impacting the fitness of subsequent generations.

## Results

### Downregulation of motif-dependent piRNA biogenesis in response to increased temperature

**Fig. 1:**
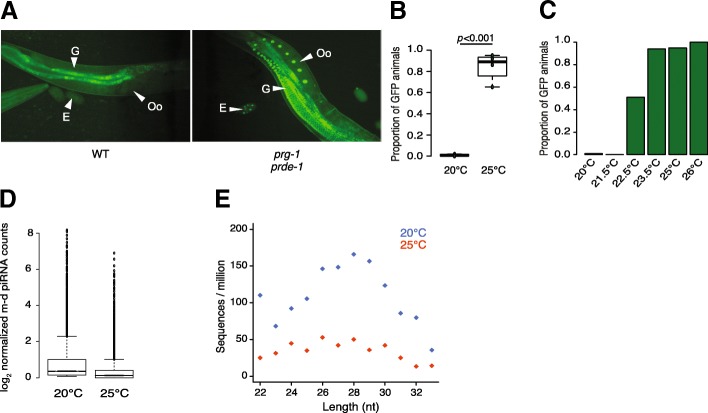
Reduction in motif-dependent piRNA biogenesis at increased temperature. **a** Expression of GFP in nuclei of the gonad (G), oocytes (Oo) and eggs (E) of animals carrying the piRNA sensor at 20 °C in the wild-type strain (WT, left), and in mutants for the piRNA pathway (*prg-1* and *prde-1*, right). **b** Proportion of wild-type animals expressing GFP at 20 °C and 25 °C. At least 30 worms have been screened for each conditions in each of the 4 replicates (20 °C *n*_total_ = 138, 25 °C *n*_total_ = 208, two-sided Student’s *t* test). Animals were grown at the tested temperature for their entire developmental cycle (i.e. from fertilisation to adulthood). **c** Proportion of wild-type animals expressing GFP at 20 °C (*n* = 75), 21.5 °C (*n* = 50), 22.5 °C (*n* = 45), 25 °C (*n* = 89) and 26 °C (*n* = 49). **d** Log_2_ motif-dependent piRNA counts normalised to the total number small RNAs in the wild-type strain at 20 °C and 25 °C. Animals were grown at the tested temperature from the first larval stage to adulthood. 18,571 piRNA loci with a motif score > 7 according to the algorithm used in Ruby et al. [15] were examined. **e** Abundance of piRNA precursors (26 to 30 nt) in the wild-type strain at 20 °C and 25 °C. piRNA precursors from any of the 18,571 loci as above were examined

To test the effect of environmental conditions on piRNA-mediated silencing, we used a strain of *C. elegans* with a germline-expressed GFP transgene with a perfect target site for a specific motif-dependant piRNA in its 3′UTR [22]. The piRNA sensor is silenced in wild-type worms at 20 °C whilst mutants lacking either *prde-1* or *prg-1* desilence the piRNA sensor at 20 °C, indicating that the activity of the piRNA pathway is required for sensor silencing [23] (Fig. 1a).

Whilst the piRNA sensor is completely silenced in wild-type animals at 20 °C, when grown at 25 °C, the GFP signal became visible in most animals (*p* < 0.001) indicating desilencing of the piRNA sensor (Fig. 1b). A temperature gradient between 20 °C and 26 °C indicated a switch in piRNA silencing above 22.5 °C above which notable silencing could be observed (Fig. 1c). Expression of the piRNA sensor was not increased by temperature in *prde-1* or *prg-1* mutants that lack a functional piRNA pathway, thus confirming that the increased expression of the sensor is due to compromised piRNA-mediated silencing at higher temperature.

To investigate the mechanism of reduced piRNA silencing, we undertook small RNA sequencing from animals grown at 20 °C or 25 °C. We used standard hypochlorite treatment to obtain large numbers of embryos and after hatching to L1s at 20 °C, we grew animals to young adult stage at either 20 °C or 25 °C before isolating RNA for small RNA library preparation (see the section “Methods”). Mature piRNA levels, normalised to total 18–33 nucleotide long small RNAs (see the section “Methods”), derived from motif-dependent loci were reduced at 25 °C (*p* < 1e−16, Wilcox unpaired test; Fig. 1d). Competition between small RNA pathways in *C. elegans* has been well documented [24–26]. We therefore investigated how other small RNA pathways respond to temperature. 22G-RNAs showed no change at 25 °C relative to 20 °C (Additional file 1: Figure S1A). miRNAs showed a small but significant increase at 25 °C (Additional file 1: Figure S1B); however, the change was less statistically significant than the reduction in piRNAs (Additional file 1: Figure S1A). Additionally, the reduction in motif-dependent piRNAs was robust to different normalisation methods (Additional file 1: Figure S1C). Thus, we conclude that the increased temperature likely affects the piRNA pathway directly.

We next investigated the mechanism accounting for reduced mature piRNA levels. One possible mechanism would be reduced *prg-1* protein levels, which has been observed previously at 25 °C [13]. However, since *prg-1* protein is reduced in the absence of 21Us [23], this could be a consequence rather than a cause of reduced piRNAs. Consistent with this interpretation, we saw no consistent change in *prg-1* transcript levels at 25 °C relative to 20 °C (Additional file 2: Figure S2E). An alternative explanation for reduced piRNA levels could be an effect on piRNA biogenesis. We therefore investigated piRNA precursors, which are capped 26–30 nt long small RNAs with a 2′ 5-extension relative to the mature 21U-RNA [17, 18, 27]. piRNA precursors were strongly reduced at 25 °C (Fig. 1e). Moreover, motif-independent piRNAs, which bind to *prg-1* but which are produced independently of *prde-1* [17, 18, 28, 29], were not significantly reduced at 25 °C (Additional file 1: Figure S1D). Importantly, we tested whether this was due to reduced power to detect differences in motif-independent piRNAs due to their lower abundance by downsampling motif-dependent piRNA numbers to the number of motif-independent piRNAs detected. We detected a significant reduction (*p* < 1e−16) in motif-dependent piRNAs in all samples and the difference was significantly larger than the difference in motif-independent piRNAs in every sample (Additional file 1: Figure S1E and F). Thus, we conclude that the reduction in motif-dependent piRNAs at 25 °C is due to reduced piRNA biogenesis. Intriguingly, we observed the same response in piRNA biogenesis for two other nematode species *Pristionchus pacificus* (Additional file 2: Figure S2A and B) and *Caenorhabditis briggsae* (Additional file 2: Figure S2C) when shifted from 20 to 25 °C, despite the fact that *C. briggsae* has a higher optimal growth temperature than *C. elegans* and suggesting that the temperature-dependent response is widely conserved.

### Temperature induces intergenerational reduction in piRNA-mediated silencing

**Fig. 2:**
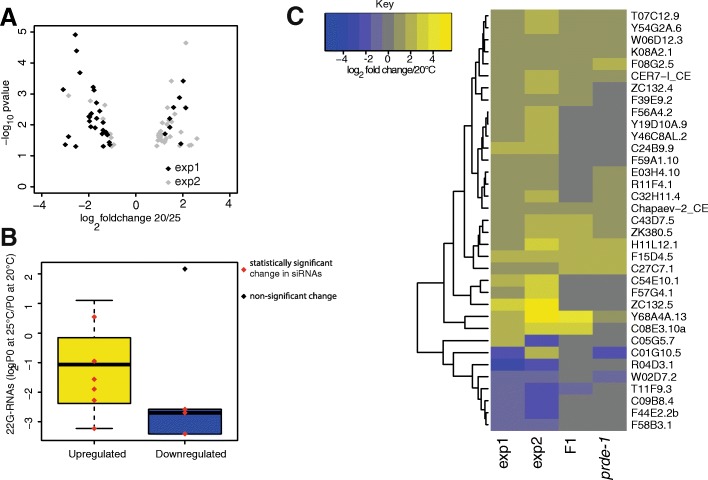
Intergenerational gene expression alterations induced by increased temperature. **a** Volcano plot presenting the overlap of downregulated genes (Log_2_ fold change 20 °C/25 °C < 0) and upregulated genes (Log_2_ fold change 20 °C/25 °C > 0) at 25 °C between adults P0 animals (exp1) and the time course experiment (exp2). **b** Changes in secondary piRNA production for upregulated genes (yellow) and downregulated genes (blue) at 25 °C compared to 20 °C for the overlapping genes from exp1 and exp2. **c** Heatmap representing the behaviour of these genes for P0 at 25 °C (exp1), the time course experiment at 25 °C (exp2), F1 grown at 20 °C from P0 grown at 25 °C (F1) and *prde-1* mutant animals. Numbers of genes involved are shown in Additional file 4: Figure S3A, B and C

The ability of environmental perturbations to affect the piRNA pathway provides an example whereby environmental conditions can be either sensed directly by the germline or communicated to the germline from the soma. As piRNAs can initiate transgenerational epigenetic alterations, the effects of perturbations may remain once the environmental perturbations have been removed. To test this, we used RNAseq to investigate gene expression in animals grown at either 20 °C or 25 °C. We first isolated RNA from nematodes at the onset of egg-laying at both temperatures and identified differentially expressed genes. However, many *C. elegans* genes oscillate throughout developmental time, and the changes we detected were dominated by changes in such variable genes suggesting that subtle differences in developmental stage between animals grown at different temperatures might be responsible for these changes. In order to avoid this, we isolated RNA in duplicate from young adult worms 8, 16 and 24 h after the visually distinctive fourth larval moult (L4 stage) at both 20 °C and 25 °C and searched for gene expression changes that were consistently regulated across all three timepoints. This identified 37 genes that were consistently affected across different timepoints (Fig. 2a; Additional file 3: Table S1; Additional file 4: Figure S3a).

To test the involvement of the piRNA pathway, we sequenced small RNAs from *C. elegans* grown at 20 °C or 25 °C. Direct targets of piRNAs are complicated to predict due to imperfect complementarity [10, 22] and the possibility of transgenerational epigenetic memory meaning that certain genes are no longer dependent on piRNA recognition [20, 21]. We therefore mapped 22G-RNAs, the effectors of piRNA-mediated silencing, to genes [22] and examined the changes in small RNAs on genes with consistent expression changes at 25 °C relative to 20 °C. Genes that were consistently upregulated all showed a significant decrease in small RNA levels (*p* < 0.01; Fig. 2b). However, genes that were downregulated did not show upregulated small RNAs. This suggested that loss of piRNA-mediated silencing might result in upregulation of genes due to loss of 22G-RNAs, but that downregulation of genes was not a direct result of piRNA changes.

To further examine the role of the piRNA pathway in temperature-mediated gene expression changes, we compared the set of genes identified with gene expression changes identified in *prde-1* mutants lacking motif-dependent piRNAs. Genes upregulated at 25 °C showed a statistically significant overlap with genes upregulated in *prde-1* mutants at 20 °C relative to N2 (Fig. 2c and Additional file 4: Figure S3B), consistent with downregulation of piRNAs at increased temperature (Fig. 1).

We next investigated gene expression changes in the progeny of animals raised at either 25 °C or 20 °C after return to 20 °C. There was significant overlap between genes upregulated at 25 °C in P0 animals and genes upregulated in F1 animals derived from parents grown at 25 °C (Fig. 2c and Additional file 4: Figure S3C). The majority of these shared genes were also upregulated in *prde-1* mutants (Fig. 2c and Additional file 4: Figure S3C), supporting a causal role for the piRNA pathway in the regulation of these genes.

### Intergenerational epigenetic inheritance influences adaptation to temperature

**Fig. 3:**
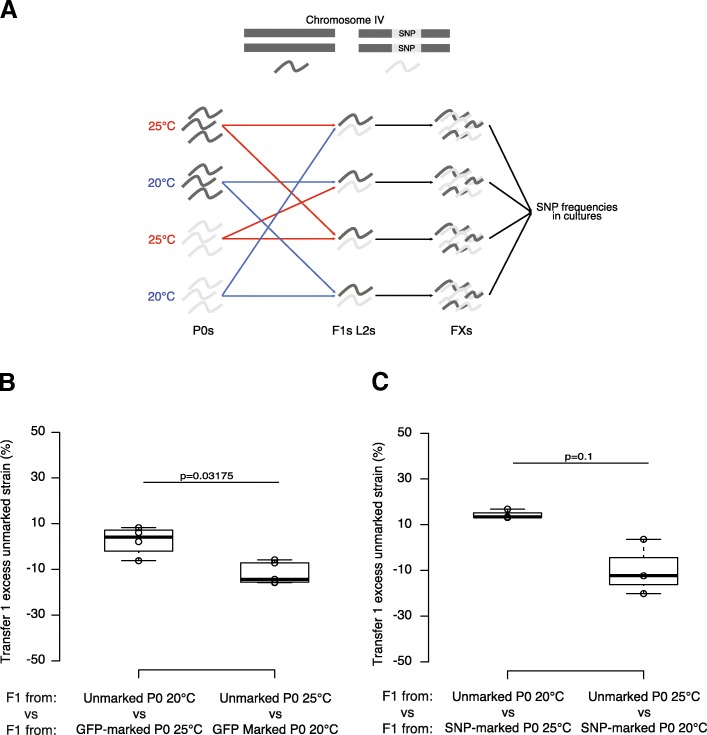
Intergenerational fitness effects of growth at altered temperatures. **a** Experimental design used for the competition assay. Two strains with either different fluorescence (unmarked or GFP-marked) or different genotypes (N2 or N2 containing introgressed SNPs from JU1580 on Chromosome IV) were grown at 20 °C or 25 °C and then synchronised using sodium hypochlorite treatment (bleaching). Equal number of F1s from two different parental treatments was then put in the same plate. All different combinations have been tested in parallel either in triplicate or in quintuplicate. Before starvation, animals were harvested and the proportions of each of the different strains measured using a fluorescent microscope (GFP-marked animals) or using pyrosequencing (SNP-marked animals). 200 to 400 animals were transferred to a fresh NGM plate and the proportions were measured similarly. **b** Intergenerational competition at 20 °C between animals derived from (left) unmarked P0 animals grown at 20 °C and GFP-marked P0 animals grown at 25 °C; (right) GFP-marked P0 animals grown at 20 °C and unmarked P0 animals grown at 25 °C. **c** Competition at 20 °C between animals derived from (left) unmarked P0 animals grown at 20 °C and SNP-marked P0 animals grown at 25 °C; (right) SNP-marked P0 animals grown at 20 °C and unmarked P0 animals grown at 25 °C. *Y*-axes show the proportion of unmarked animals after 1 transfer for all replicates, corrected for the effect of the SNP or the GFP by subtracting the mean of the proportions of animals grown at 20 °C when both P0 strains were grown at 25 °C. The raw data are available in Additional file 5: Table S2

To investigate potential consequences of transgenerational epigenetic changes induced in response to temperature, we carried out competition experiments at 20 °C between the progeny of animals grown at 20 °C or 25 °C, using either an introgression from the *C. elegans* strain JU1580 **(***mfIR41(IV,JU1580>N2),* using a single nucleotide polymorphism (SNP) WBVar01650008 as a marker**)** into the laboratory strain N2 or a GFP as markers (Fig. 3a and Additional file 5: Table S2). F1 animals derived from parents grown at 20 °C outcompeted animals from parents grown at 25 °C in both experiments (Wilcox test; *p* < 0.05 for F2/3 Fig. 3b; *p* = 0.1, smallest *p* value possible for *n* = 3, for F2/3 Fig. 3c, and Additional file 5: Table S2, also see Additional file 6: Figure S4C for further generations). However, F1 animals derived from parents grown at 25 °C do not outcompete F1 animals derived from parents grown at 20 °C when F1s are grown at 25 °C (Wilcox test; *p* > 0.5 for F2/3, Additional file 6: Figure S4A and B). Thus, growth at 25 °C leads to reduced fitness of subsequent generations at 20 °C without improving the fitness of subsequent generations at 25 °C, at least when grown on *E. coli* as a food source within the laboratory.

### Bacterial infection restores motif-dependent piRNA levels at increased temperatures

**Fig. 4:**
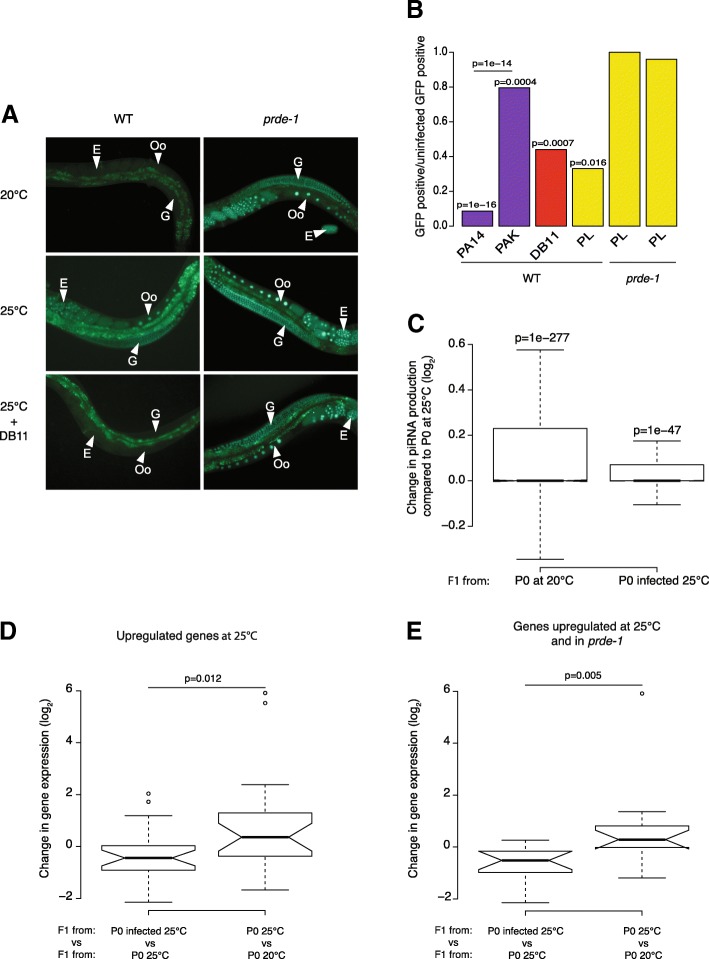
Bacterial infection suppresses reduced piRNA levels at 25 °C. **a** Expression of GFP in nuclei of the gonad (G), oocytes (Oo) and eggs (E) of animals carrying the piRNA sensor in the wild-type strain (WT, left) and in the *prde-1* mutant (*prde-1* right) both grown from hatching either at 20 °C or 25 °C on regular bacteria (HB101) or at 25 °C on the pathogenic bacteria *Serratia marcescens* (DB11). **b** Proportion of wild-type animals (WT) and *prde-1* animals expressing GFP on: *P. aeruginosa* (purple) PA14 (high toxicity, *n* = 47) and PAK (low toxicity, *n* = 73); *S. marcescens* DB11 (red, *n* = 106); and *P. luminescens* (yellow; WT *n* = 31; *prg-1 n* = 28; *prde-1 n* = 46) relative to the proportion of GFP-positive animals grown at 25 °C on regular food (*n* = 38). Fisher’s exact test was used to evaluate differences between conditions. Raw data are available in Additional file 9: Table S4. **c** Change (Log_2_) in mature piRNA levels in F1s from parents grown at 20 °C (P0 at 20 °C) or grown at 25 °C (P0 at 25 °C) in presence of *Serratia marcescens* compared to F1s from parents grown at 25 °C. **d**, **e** Changes (Log_2_) in gene expression in F1s from infected P0s compared to F1s from P0s grown at 25 °C (left) and F1s from P0s grown at 25 °C compared to F1s from P0s grown at 25 °C (right) for: (**d**) genes previously identified as intergenerationally upregulated when parents grown at 25 °C; (**e**) genes intergenerationally upregulated both when parents were grown at 25 °C and in the *prde-1* mutant

Whilst undergoing experiments to test the effect of temperature on piRNA silencing, we noticed anecdotally that occasional contamination of plates with non-*E. coli* bacteria, which impaired growth of *C. elegans*, resulted in lack of induction of the sensor at 25 °C. miRNAs have been implicated in pathogen responses [30, 31] and, anecdotally, to impact on knockdown of genes by RNAi [32]. We therefore wondered whether infection of nematodes with pathogenic bacteria had any effect on silencing by the piRNA pathway. *C. elegans* has been used frequently to model infection with pathogenic bacteria; we therefore decided to use the piRNA sensor to test whether such infections had any affect on piRNA-mediated silencing. We infected animals grown at 25 °C with virulent *Pseudomonas aeruginosa* (PA14) or an attenuated strain (PAK) and compared to animals grown on *E. coli*. Whilst the sensor was induced as expected at 25 °C when grown on PAK or *E.coli*, animals grown on PA14 retained piRNA sensor silencing (*p* < 0.05, Fisher’s exact test Fig. 4a, b, Additional file 7: Table S3). Similarly, infection with other known *C. elegans* bacterial pathogens *Serratia marcescens* (DB11) and the less virulent *Photorhabdus luminescens* (PL), resulted in increased piRNA sensor silencing relative to uninfected control animals (both *p* < 0.05, Fisher’s exact test; Fig. 4b). The increased silencing of the piRNA sensor induced by infection was dependent on the piRNA pathway because infection of either *prg-1* or *prde-1* did not lead to sensor silencing (Fig. 4a, b).

We next sequenced piRNAs from F1 animals grown at 20 °C derived from parents either grown at 20 °C, 25 °C or at 25 °C on *S. marcescens*. Importantly, F1 animals derived from parents grown at 25 °C show reduced levels of mature piRNAs compared to F1 animals derived from parents grown at 20 °C (Fig. 4c). This suggests that the reduction in piRNAs observed as a result of growth at 25 °C persists in their progeny despite return to 20 °C. However, mature piRNA levels were higher in F1 animals derived from parents infected with *S. marcescens* than from uninfected parents, indicating that growth at 25 °C with *S. marcescens* suppressed the reduction in piRNA levels in F1 animals (*p* < 0.001, Wilcoxon paired test).

To test how this related to changes in gene expression, we carried out RNAseq on the F1 animals derived from infected animals grown at 25 °C and compared to F1 animals derived from uninfected controls grown at 25 °C. Both sets of F1 animals were returned to 20 °C. Genes previously identified as upregulated at 25 °C (Fig. 2) remained upregulated in F1 animals derived from uninfected controls (Fig. 4d), consistent with the intergenerational effect of gene expression changes that we observed previously (Fig. 2) and consistent with our observation that piRNA levels are reduced in these F1 animals. However, genes upregulated at 25 °C were no longer upregulated in progeny of animals infected with *S. marcescens*. Thus, bacterial infection suppressed intergenerational changes in gene expression. Importantly, the effect was statistically stronger in the subset of temperature-sensitive genes that are also upregulated in *prde-1* animals than in genes that are not upregulated in *prde-1* (Fig. 4e, Additional file 8: S5A and S5B), suggesting that the intergenerational upregulation of these genes is due to reduced piRNA levels.

Taken together, our results demonstrate that bacterial infection suppresses intergenerational gene expression changes induced by growth of parent animals at 25 °C by suppressing the reduction in piRNA levels that occurs at this temperature.

### Bacterial infection improves fitness intergenerationally

**Fig. 5:**
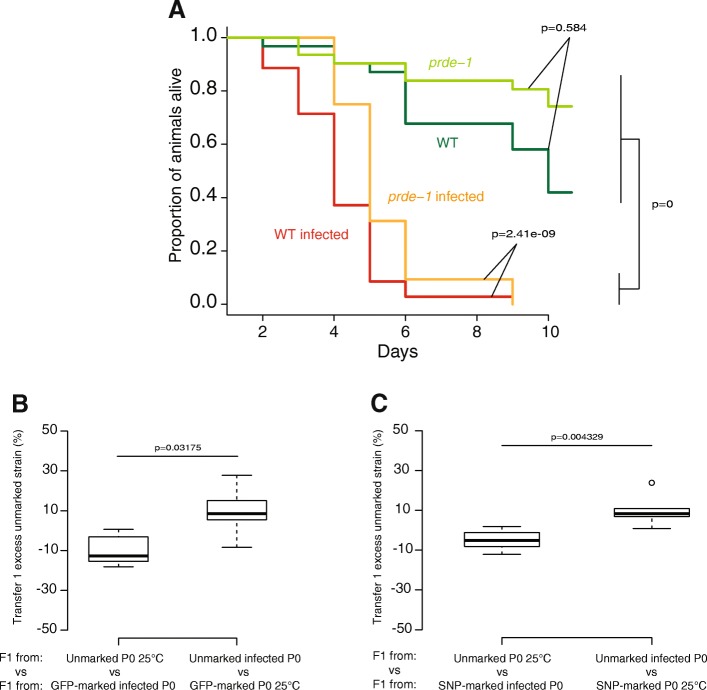
Bacterial infection improves fitness in subsequent generations. **a** Survival of the wild-type strain (WT) and the *prde-1* mutant at 25 °C on regular food (dark green and light green) and on the pathogenic bacteria *Serratia marcescens* (red and orange). A Mantel-Haenszel Logrank test has been used to assess the significativity between the different treatments. Raw data are available in Additional file 9: Table S4. **b** Intergenerational competition at 20 °C between animals derived from (left) unmarked P0 animals grown on *E. coli* at 25 °C and GFP-marked animals grown on S*. marcescens* at 25 °C; (right) GFP-marked P0 animals grown on *E. coli* at 25 °C and unmarked animals grown on *S. marcescens* at 25 °C. **c** Intergenerational competition at 20 °C between animals derived from (left) unmarked P0 animals grown on *E. coli* at 25 °C and SNP-marked P0 animals grown on *S. marcescens* at 25 °C; (right) SNP-marked P0 animals grown on *E. coli* at 25 °C and unmarked P0 animals grown on *S. marcescens* at 25 °C. *Y*-axes for **b** and **c** show the percentage of unmarked animals after 2–3 generations (1 transfer, all biological replicates), corrected for the effect of the SNP or GFP marker as in Fig. 3. Raw data are available in Additional file 5: Table S2

We wondered what the functional consequences of piRNA pathway modulation by bacterial infection might be. We first tested whether increased piRNA levels induced by bacterial infection might affect susceptibility to infection. *Prde-1* mutants, which lack motif-dependent piRNAs [17], showed the same sensitivity to infection with *S. marcescens* as wild-type *C. elegans*, indicating that piRNAs do not directly contribute to resistance to this pathogen (*p* > 0.1 Maental-Hutzel log test for interaction between *prde-1* and *S. marcescens*; Fig. 5a and Additional file 9: Table S4). Since bacterial infection suppresses temperature-dependent intergenerational changes in piRNAs and gene expression, we hypothesised that bacterial infection might similarly suppress intergenerational fitness defects resulting from growth at 25 °C. Consistent with this hypothesis, progeny of DB11-infected animals outcompeted progeny of uninfected animals when both were grown at 20 °C (Fig. 5b, c, *p* < 0.05, Wilcoxon unpaired test, further generations see Additional file 6: Figure S4D). Thus, the fitness of animals is improved by infection of the previous generation with pathogenic bacteria.

## Discussion

**Fig. 6:**
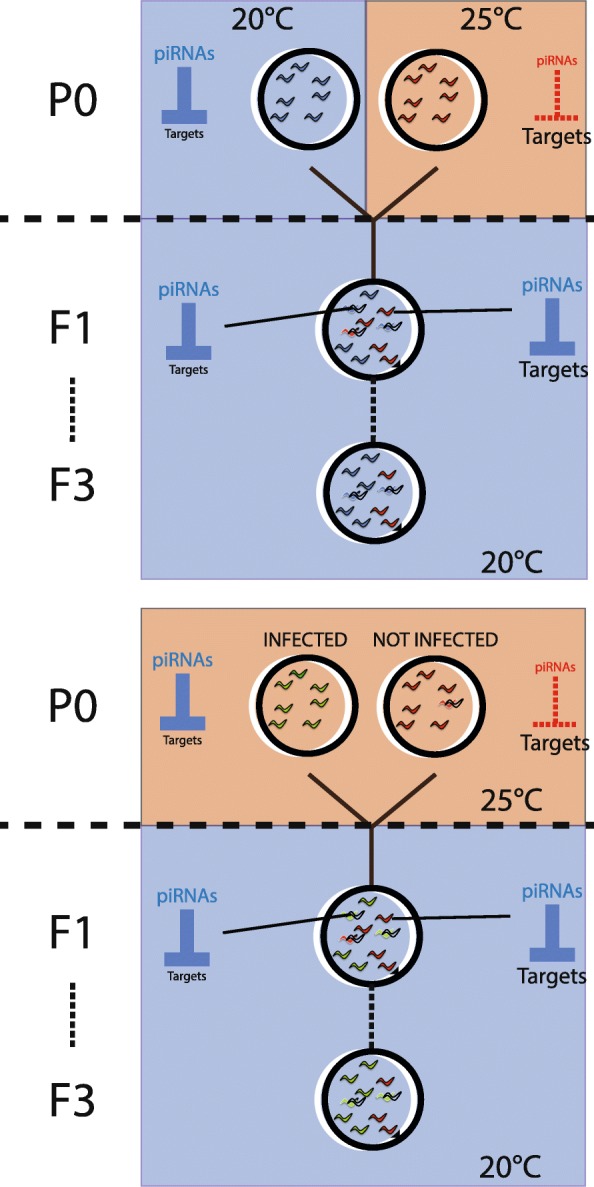
Environmental conditions affect piRNA biogenesis and fitness intergenerationally in *C. elegans*. PiRNA biogenesis is reduced at 25 °C compared to 20 °C leading to an increase of piRNA targeted genes. The increase of expression of certain genes observed in parental animals grown at 25 °C and resulting from the loss of piRNAs is maintained in the progeny even if grown at 20 °C. Descendants of animals grown at 25 °C display a decrease in fitness at 20 °C compared to descendants of animals grown at 20 °C or animals grown at 25 °C on pathogenic bacteria

Despite much interest in multi-generational epigenetic phenomena in many model systems, including *C. elegans*, little is known of what role this might have in the lifecycle of animals in the wild. Our study offers two important new insights into this, first by demonstrating that environmental conditions modulate the piRNA pathway in the germline to establish gene expression changes both in animals exposed to the perturbation and in their offspring, and second by showing that gene expression changes induced in this manner influence fitness over a limited number of generations (Fig. 6).

### Modulation of *C. elegans* piRNA biogenesis in response to the environment

Environmental effects on the effects of small RNA pathways in *C. elegans* have been observed previously (e.g. [4, 5, 33]). Our study shows for the first time that, despite the constitutive requirement for the piRNA pathway in normal germline function, environmental conditions can directly modulate piRNA biogenesis. Interestingly, our work shows that different stresses (temperature and bacterial infection) have opposite effects on piRNA levels, raising the possibility that there exist mechanisms to directly signal the effect of environmental changes to the piRNA biogenesis machinery. It is possible that the germline may directly sense changes in temperature to modulate piRNA biogenesis. However, importantly, the pathogenic bacteria we studied infect the *C. elegans* gut [34], implying the existence of a mechanism to transmit signals between the soma and the germline. The identity of this signalling method is still unclear; it does not appear to involve the canonical stress-signalling axis in *C. elegans* as *pmk-1* mutants do not show a difference in mature piRNA levels (Additional file 2: Figure S2D). Establishing the identity of any such sensing mechanism is an interesting prospect for future work; its existence would additionally strengthen the possibility that stress responses could regulate piRNA biogenesis in other species.

### Intergenerational epigenetic inheritance in the environment

Our finding that environmental stimuli can modulate the piRNA pathway provides important new insights into the elusive functions of multi-generational epigenetic inheritance in *C. elegans*. We demonstrate that a moderate increase in temperature leads to loss of motif-dependent piRNA silencing. Some of these changes result in sustained gene expression alterations and also resulted in a loss of fitness in subsequent generations returned to 20 °C. To our knowledge, this is the first direct demonstration of an intergenerational effect in *C. elegans* that has a fitness consequence under physiological conditions.

The reduced fitness at 20 °C as a result of parental exposure to higher temperature appears to be limited to around 1–3 generations post exposure (Fig. 3 and Additional file 6: S4). Nevertheless, it seems surprising that natural selection has not eliminated this effect. The N2 laboratory strain that we used for our experiments has adapted to the laboratory under relatively constant conditions over multiple generations; thus, it is possible that the responses we observe are laboratory adaptations specific for animals mostly raised at constant temperature. Our observation that similar temperature-dependent responses in several strains of *C. briggsae* and in *P. pacificus* (Additional file 2: Figure S2), both of which were introduced into the laboratory much more recently than N2, argues that this is not a *C. elegans*-specific laboratory adaptation. Instead, we suggest that an interesting possible explanation is raised by our observation that bacterial infection suppresses the temperature-dependent reduction in piRNA levels and leads to intergenerational increase in fitness relative to uninfected animals. We propose that in the natural environment of *C. elegans*, raised temperature will increase the growth rate of bacteria, many of which will be pathogenic to *C. elegans* [35]. Thus, in the natural environment of *C. elegans*, increased temperature may usually be accompanied by increased infection, so the intergenerational fitness cost of growth at higher temperature is never visible to natural selection. This suggests that *C. elegans* is adapted to chronic pathogen infection, representing a nematode equivalent of the famous “hygiene hypothesis” used to explain the incidence of allergy and autoimmune diseases in human populations [36]. It also presents the possibility that many other multi-generational epigenetic effects may be the result of the artificial environment of the organism under laboratory conditions and not usually visible to natural selection in wild populations.

## Conclusion

Here, we show that the *C. elegans* piRNA pathway responds to alterations in environmental conditions. Temperature decreases piRNA levels but bacterial infection increases it, and these alterations set up gene expression changes that persist into the next generation despite removal of the initial environmental stimuli. Importantly, at least in the case of bacterial infection, it is likely that the environmental effect on the piRNA pathway must be mediated by signalling from the soma to the germline. Additionally, we show, for the first time, potential fitness consequences of the dynamic changes in piRNA levels. Our work acts as a starting point for further exploration of the role of multi-generational epigenetic changes for *C. elegans* in its natural environment.

## Methods

### Nematode strains

The N2 strain of *Caenorhabditis elegans* is considered as the wild-type (WT) strain.

The strain SX1316 (mjIs144 II) expresses a piRNA sensor which is off in presence of specific piRNAs and on in absence of those piRNAs. The strains SX1888 and SX2470 also express this sensor but carry *prg-1(n4357)I* and *prde-1(mj207)V*, respectively. The strain SX2499 carries the *prde-1(mj207)V* mutation. SX328, used for the completion assay, carries the transgene mjIs17 IV resulting in the expression of GFP in the pharynx. JU2196 is an introgression of the centre of Chromosome IV from the wild isolate JU1580 into the WT background (*mfIR41(IV,JU1580>N2*). Animals were grown on NGM plates with *Escherichia coli* HB101.

*Pristionchus pacificus* (PS312) and *Caenorhabditis briggsae* (AF16) were maintained on HB101 on NGM plates.

SX328 was obtained from CGC.

### Bacterial strains used for infections

#### *Pseudomonas aeruginosa*

PA14 and PAK were used as infectious and non-infectious strains, respectively. Each strain was first streaked onto a 10-cm LB agar plate and incubated at 37 °C for 12 to 16 h. We then inoculated a single colony into 5 ml LB broth and incubated at 37 °C with aeration for 12 to 16 h. We seeded 250 μl of the culture on specific Slow Killing plates and incubated them at 37 °C for 24 h. We finally transferred the plates to 25 °C for 24 h before using them.

#### *Photorabdus luminescens*

Preparation of plates was as for *P. aeruginosa* except 30 °C was used instead of 37 °C and the final seeding was made onto NGM plates.

#### *Serratia marcescens*

DB11 was used as an infectious strain. It was first streaked onto a 10-cm LB agar plate and grown at 37 °C overnight. We then inoculated a single colony into 5 ml LB broth and incubated at 37 °C with aeration for 12 to 16 h. We seeded 100 μl of the culture on NGM plates and incubated them at 37 °C Overnight. We finally transferred the plates to 25 °C overnight before using them.

### Temperature and infection assays

#### Basic temperature assay

For visual examination of sensor silencing, ~ 5–10 L4 animals were put onto plates at either 20 °C or 25 °C and left for 16 h to lay embryos. Adults were then removed, leaving embryos to hatch and reach adulthood. Animals were then mounted onto slides, and GFP was visualised using a fluorescent microscope.

For sequencing experiments, populations were synchronised by extracting embryos using sodium hypochlorite treatment (“bleaching”) and hatching embryos to L1s overnight in M9 medium. Between 100 and 200 L1s were placed on NGM plates at 20 °C or 25 °C. Once they reached early adulthood, animals were harvested and washed with M9 for RNA sequencing experiments.

### Intergenerational temperature assay

Populations were synchronised by extracting embryos using sodium hypochlorite treatment (“bleaching”) and hatching embryos to L1s overnight in M9 medium. Two plates containing 100 to 200 arrested L1s were put at 20 °C or 25 °C. Once animals reached early adulthood, they were harvested, washed three times with M9, and either RNA was extracted or animals were treated with hypochlorite solution. L1s resulting from the hypochlorite treatment were then grown at 20 °C and collected as young adults.

### Basic infection assays

Populations were synchronised by extracting embryos using sodium hypochlorite treatment (“bleaching”) and by hatching embryos to L1s overnight in M9 medium. Between 100 and 200 L1s were placed on NGM plates at 20 °C, 25 °C or at 25 °C in the presence of *Serratia marcescens, Pseudomonas aeruginosa* or *Photorabdus luminescens*. Once they reached early adulthood, animals were either mounted on slides and observed using a fluorescent microscope or were harvested and washed with M9 for further RNA sequencing experiments.

### Intergenerational infection assay

Populations were synchronised by extracting embryos using sodium hypochlorite treatment (“bleaching”) and by hatching embryos to L1s overnight in M9 medium. Two plates containing 100 to 200 arrested L1s were put at 20 °C, 25 °C or at 25 °C in the presence of *Serratia marcescens*. Once animals reached early adulthood, they were harvested, washed three times with M9, and either RNA was extracted or animals were treated with hypochlorite solution. L1s resulting from the hypochlorite treatment were then grown at 20 °C and collected as young adults.

### Time course temperature assay

Cultures were synchronised as previously described. Between 100 and 200 arrested L1s were then plated onto NGM plates at 20 °C. Once they reached adulthood, animals were treated once more with hypochlorite solution. Arrested L1s resulting from hypochlorite treatment of adults were plated onto NGM plates and grown either at 20 °C or 25 °C. Upon reaching early L4 stage, animals were collected in M9 every 2 h for the treatment at 20 °C or every 1.5 h for the treatment at 25 °C. Collected animals were then washed and put into Trizol before RNA extraction and small RNA library preparation.

### Competition assays

The competition assay was performed as described [37] with minor modifications. For each different experiments, each of the two tested strains’ embryos (SX238 vs N2 or N2 vs JU2196) were extracted using hypochlorite treatment (“bleaching”) and hatched overnight in M9 medium to give synchronised L1 larvae. L1s were then plated on each of the tested conditions in parallel (20 °C vs 25 °C or 25 °C vs DB11 infection) to give the starting population (P0). One day after they reached adulthood, embryos were isolated by bleaching, hatched to L1s overnight and plated onto the final condition of the competition to give the F1 generation. To initiate the competition assay, 10 progeny from each P0 condition (F1s) were put to compete with 10 F1s of the other strain from the same or a different parental condition so that each combination of two strains and two parental conditions were represented. Five days later and every 2 days subsequently, animals were collected from plates in M9 and 200–400 transferred onto a new NGM plate. The remaining animals were frozen in M9 at − 20 °C for subsequent analysis of SNP proportions (WBVar01650008), or analysed for fluorescent protein expression by microscopy. Thus, the sample taken at transfer 1 corresponds to a mixture of generations F2 and F3 and samples at transfer 2 corresponds to a mixture of generations F3 and F4. Sample sizes for the fluorescent protein analysis are available in Additional file 5: Table S2.

The proportion of each SNP was assessed by pyrosequencing, using a PyroMark Q96 ID instrument from Biotage (Uppsala, Sweden). Five microlitres of the frozen sample was mixed for a Worm Lysis into 45 μl of buffer containing proteinase K at 100 μg/ml. In order to verify whether the measurement of proportions were correct, we also prepared lysis containing different proportions of the two parental strains (100%, 80%, 60%, 50%, 40%, 20% and 0%). We added 2 μl of worm lysate to 23 μl of PCR mix composed of 12.5 μl of Quick-Load® Taq 2X Master Mix from NEB, 0.1 μl of non-biotinylated reverse primer (cgccagggttttcccagtcacgacattctgcaacaaaaaaaattaagc) at 10 mM, 0.4 μl of corresponding universal biotinylated primers (biotin-cgccagggttttcccagtcacgac) at 10 mM, and 0.5 μl of the forward primer (accctaaaaaaacccgataaaatt) at 10 nM. Single-stranded DNA was then purified, and the pyrosequencing reaction was performed following the manufacturer’s indications using the appropriate sequencing primer (cagtaaagtctcaacaaatg).

### Data analysis

Both strain combinations have minor differences in baseline fitness. We thus corrected for this by subtracting the proportion of N2 when competing at 25 °C. The corrected proportion of N2 was then tabulated alongside growth condition of parental strain (infection or treatment with elevated temperature). We then compared conditions using an unpaired two-sample Wilcoxon test. Raw data is available as supplemental Additional file 5: Table S2.

### Longevity assay on *S. marcescens*

N2 and SX2499 cultures were synchronised with hypochlorite solution. L1s were grown at 25 °C until they reached early L4 stage. Ten animals were transferred into five plates containing HB101 and five plates containing DB11 for a total of 50 animals per treatment per strain. Every day for 10 days, each animal’s death was reported. Animals that disappeared or were found stuck on the plastic border of the plate were excluded from the analysis. During the egg-laying period, animals were transferred to a new plate every day. We used the survdiff function (Mantel-Haenszel Logrank test) from the survival package from R-Cran for statistical analysis. Raw data is available as Additional file 9: Table S4.

### RNA extraction

Once harvested, animals were washed three times in M9 and we extracted RNA using Trizol® (Invitrogen) (5–10 vol∶vol of pelleted worms). The extraction product was suspended in 20 μl in RNAse-free ddH_2_O.

### Small RNA library preparation

#### Decapping RNA

We added 1 to 5 μg of total RNA to a mix containing 1.5 μl RNA 5′ Pyrophosphohydrolase (RppH,NEB), 2 μl of NEB buffer 2 and H_2_O, up to 20 μl total reaction volume. The final mix was incubated at 37 °C for 1 h and cooled down at 4 °C for 2 min. RNA was then purified using a phenol-chloroform extraction protocol.

### Adapter ligation, retrotranscription, amplification and purification steps

We used the TruSeq® Small RNA Library Preparation Kits from Illumina, and adapter ligation, retrotranscription, amplification and purification were performed following the manufacturer’s indication. The quantity and the quality of the libraries were then evaluated using Qubit® technology from Qiagen and the Tape Station® technology from Agilent Technologies.

### Data analysis

Fastq files of small RNAs were processed and mapped to piRNAs using exact matches and identifying piRNA precursors with extensions at the 5′ and 3′ end using custom perl scripts, as described previously [17]. piRNA loci in *P. pacificus* and *C. briggsae* were predicted by aligning small RNA sequencing reads to the respective genomes (WB235) and identifying 21U-RNAs with a GTTTC sequence between 40 and 50 nucleotides upstream. Statistical tests of differences in reads from piRNA loci were performed using the Wilcoxon paired test with two tails, as this does not assume normal distributions. To compare motif-dependent and motif-independent piRNAs whilst controlling for the different numbers of loci (Additional file 1: Figure S1D and E), > 500 random samples of motif-independent piRNA loci using the R function *sample* were made with the size of the sample equal to the number of motif-independent loci detected with at least one read in at least one sequencing run. We then compared reads within this subsample at 20 °C versus 25 °C to test whether we observed a reduction at 25 °C using a Wilcoxon unpaired test with one tail (as we are expecting the sample to approximate to the observed decrease across all reads). We also compared the differences across all sampled loci to the differences across motif-independent piRNA loci testing whether the difference in sampled motif-dependent piRNAs was larger than the difference in motif-independent piRNA loci, again using a Wilcoxon unpaired test with one tail.

Mappings to miRNAs were carried out by using exact matches to the mature sequences of *C. elegans* miRNAs extracted from miRbase (www.mirbase.org) using a custom perl script. 22G RNAs were aligned to protein coding genes using bowtie, allowing for 0 mismatches and reporting only one match per read. DESeq was used to normalise data across replicates and identify statistically significant differences using the negative binomial test [38].

### RNA sequencing

Paired-end non-directional polyA RNAseq libraries were sequenced on an Illumina Hiseq. Reads were mapped directly to a combined file containing coding sequences (WS235) and repeat consensus sequences (Repbase) using bowtie2 and processed to give counts per sequence using a custom perl script. We used DESeq to obtain normalised counts for each library and identified genes and TEs changing significantly (*p* < 0.05) according to the negative binomial test either between two independent replicates of young adults at 20 °C and 25 °C (experiment 1) or consistently changing (*p* < 0.05) at 8, 16 and 24 h after the onset of the L4 stage between 20 °C and 25 °C (experiment 2). Genes changing significantly in both experiment 1 and experiment 2 were defined as the high-confidence set of genes for further analysis to examine how they changed in *prg-1* compared to WT (mean across 8, 16 and 24 h after the onset of the L4 stage), *prde-1* compared to WT (mean across 8, 16 and 24 h after the onset of the L4 moult) and in progeny grown at 20 °C from parents grown at 25 °C compared to in progeny grown at 20 °C from parents grown at 20 °C. Raw fastq files have been uploaded to the SRA (SRP145566).

## Additional files

Additional file 1: **Figure S1.** Change in 22G small RNA from other pathways at 25 °C. (A) Comparison of total 22G-RNAs at 20 °C and 25 °C. The *p* value is a Wilcoxon unpaired test (two tailed). (B) Comparison of miRNA reads at 20 °C and 25 °C. The *p* value is a Wilcoxon unpaired test (two tailed). (C) Change in piRNA levels between 20 °C and 25 °C normalised by the miRNA reads observed in (B). *p* value is a Wilcoxon unpaired test (two tailed). (D) Comparison of expression of motif-independent piRNA between 20 °C and 25 °C. *p* value is a Wilcoxon unpaired test (two tailed). (E) Histogram of the *p* value for a reduction in motif-dependent piRNAs at 25 °C relative to 20 °C over multiple samples of motif-dependent piRNAs with the same size as the number of motif-independent piRNAs detected. The *p* value is a Wilcoxon-unpaired test for a reduction (one tailed). (F) Histogram reporting the *p* value for the change in motif-dependent piRNAs at 25 °C relative to 20 °C being larger than the change in motif-independent piRNAs over multiple samples of motif-dependent piRNAs with the same size as the number of motif-independent piRNAs detected. The *p* value is a Wilcoxon-unpaired test for a larger difference in motif-dependent piRNAs (one tailed). (PDF 2121 kb)

Additional file 2: **Figure S2.** piRNA production in different species and in the *pmk-1* mutant. (A) Log_2_ ratio of the count of 21U small RNA in *P. pacificus* between 25 °C and 20 °C. piRNAs, identified by the presence of a conserved upstream motif, are reduced relative to all other 21U-RNAs. 4956 such loci were detected and used for analysis. (B) Difference in motif-dependent piRNA precursors at 25 °C and 20 °C in *P. pacificus.* (C) Ratio of the amount of motif-dependent piRNAs at 20 °C over 25 °C in 4 different strains of *C. briggsae*. AF16 is the standard laboratory strain. In red are tropical strains and in blue are temperate strains. 2431 such piRNA loci were detected in the reference strain AF16 and used for analysis. (D) Log_2_ reads per million of motif-dependent piRNAs in the wild-type (WT) strain and in the *pmk-1* mutant at 20 °C and 25 °C. (E) Change in *prg-1* mRNA level between 20 °C and 25 °C. (PDF 536 kb)

Additional file 3: **Table S1.** Genes identified as consistently changed at 25 °C compared to 20 °C. (PDF 368 kb)

Additional file 4: **Figure S3.** Transgenerational alterations in gene expression induced by temperature. (A) Overlap between the change in gene expression at 25 °C compared to 20 °C in P0 (experiment 1) and the change in gene expression at 25 °C compared to 20 °C during the time course experiment (experiment 2). (B) Overlap between temperature-sensitive genes (grey) identified in (A), *prde-1-*dependent genes (blue) and *prg-1*-dependent genes (pink). (C) Overlap between temperature-sensitive genes in parents (grey), altered genes in *prde-1* mutants (blue) and altered genes in F1 grown at 20 °C from parents grown at 25 °C (orange). (PDF 409 kb)

Additional file 5: **Table S2.** Proportions of the different strains from the competition experiments. (PDF 392 kb)

Additional file 6: **Figure S4.** Limited duration of temperature-dependent intergenerational fitness effects. (A) Intergenerational Competition on *E. coli* at 25 °C between animals derived from (left) P0 animals grown at 25 °C marked with GFP and unmarked P0 animals grown at 20 °C; (right) unmarked P0 animals grown at 25 °C and P0 animals grown at 20 °C marked with GFP. *Y*-axis shows the percentage of unmarked animals grown after 2–3 generations (1 transfer, 5 biological replicates per condition), corrected for the effect of GFP at 25 °C. (B) Intergenerational competition on *E. coli* at 20 °C between animals derived from (left) unmarked P0 animals grown on *E. coli* at 20 °C and GFP-marked P0 animals grown at 25 °C (right) GFP-marked P0 animals grown at 20 °C and unmarked P0 animals grown at 25 °C. Y-axis shows the percentage of unmarked animals after 2–3 generations (1 transfer, 5 biological replicates per condition), corrected for the effect of GFP at 25 °C. (C) Multi-generational competition on *E. coli* at 25 °C between animals derived from (left) unmarked P0 animals grown at 20 °C and SNP-marked parents grown at 25 °C (right) SNP-marked P0 animals grown at 20 °C and unmarked parents grown at 25 °C. Y-axis shows the percentage of unmarked animals after 3–4 generations (2 transfers, 5 biological replicates per conditions), corrected for the effect of the SNP at 25 °C. (D) Multi-generational competition on *E. coli* at 20 °C between animals derived from (left) unmarked P0 animals grown on *E. coli* at 25 °C and SNP-marked P0 animals grown on *S. marcescens* at 25 °C (right) SNP-marked P0 animals grown on *E. coli* at 25 °C and unmarked P0 animals grown on *S. marcescens* at 25C. *Y*-axis shows the percentage of unmarked animals after 3–4 generations (2 transfers, 5 biological replicates per conditions), corrected for the effect of the SNP at 25 °C. (XLSX 44 kb)

Additional file 7: **Table S3.** Raw data from infection experiments. (XLSX 45 kb)

Additional file 8: **Figure S5.** Intergenerational alterations in gene expression induced by bacterial infection. Changes in gene expression in F1 s from infected P0s compared to F1s from P0s grown at 25 °C (left) and F1s from P0s grown at 25 °C compared to F1s from P0s grown at 20 °C (right) for: (A) genes previously identified as intergenerationally down-regulated when parents grown at 25 °C; (B) genes intergenerationally upregulated when parents grown at 25 °C but not up-regulated in the *prde-1* mutant. (XLSX 40 kb)

Additional file 9: **Table S4.** Raw data from survival experiments. (XLSX 42 kb)
