# Supplementary figures and images for: The piRNA pathway responds to environmental signals to establish intergenerational adaptation to stress
Source: BMC Biol. 2018 Sep 18;16:103. doi: 10.1186/s12915-018-0571-y (PMC6145337; doi:10.1186/s12915-018-0571-y)

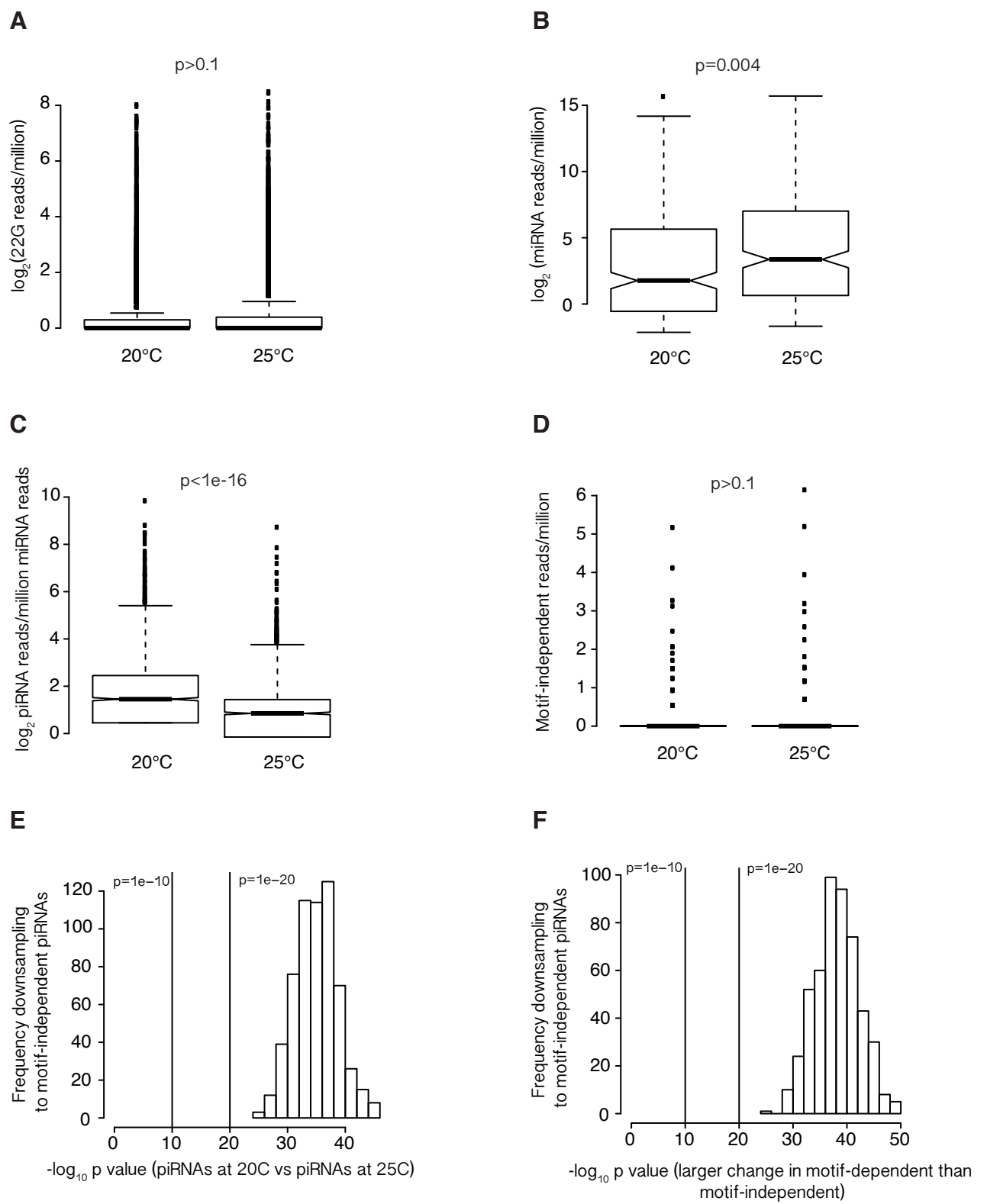

Figure S1

Supplement: Supplementary file 1 — Figure S1. Change in 22G small RNA from other pathways at 25 °C. (A) Comparison of total 22G-RNAs at 20 °C and 25 °C. The p value is a Wilcoxon unpaired test (two tailed). (B) Comparison of miRNA reads at 20 °C and 25 °C. The p value is a Wilcoxon unpaired test (two tailed). (C) Change in piRNA levels between 20 °C and 25 °C normalised by the miRNA reads observed in (B). p value is a Wilcoxon unpaired test (two tailed). (D) Comparison of expression of motif-independent piRNA between 20 °C and 25 °C. p value is a Wilcoxon unpaired test (two tailed). (E) Histogram of the p value for a reduction in motif-dependent piRNAs at 25 °C relative to 20 °C over multiple samples of motif-dependent piRNAs with the same size as the number of motif-independent piRNAs detected. The p value is a Wilcoxon-unpaired test for a reduction (one tailed). (F) Histogram reporting the p value for the change in motif-dependent piRNAs at 25 °C relative to 20 °C being larger than the change in motif-independent piRNAs over multiple samples of motif-dependent piRNAs with the same size as the number of motif-independent piRNAs detected. The p value is a Wilcoxon-unpaired test for a larger difference in motif-dependent piRNAs (one tailed). (PDF 2121 kb) [file 12915_2018_571_MOESM1_ESM.pdf]

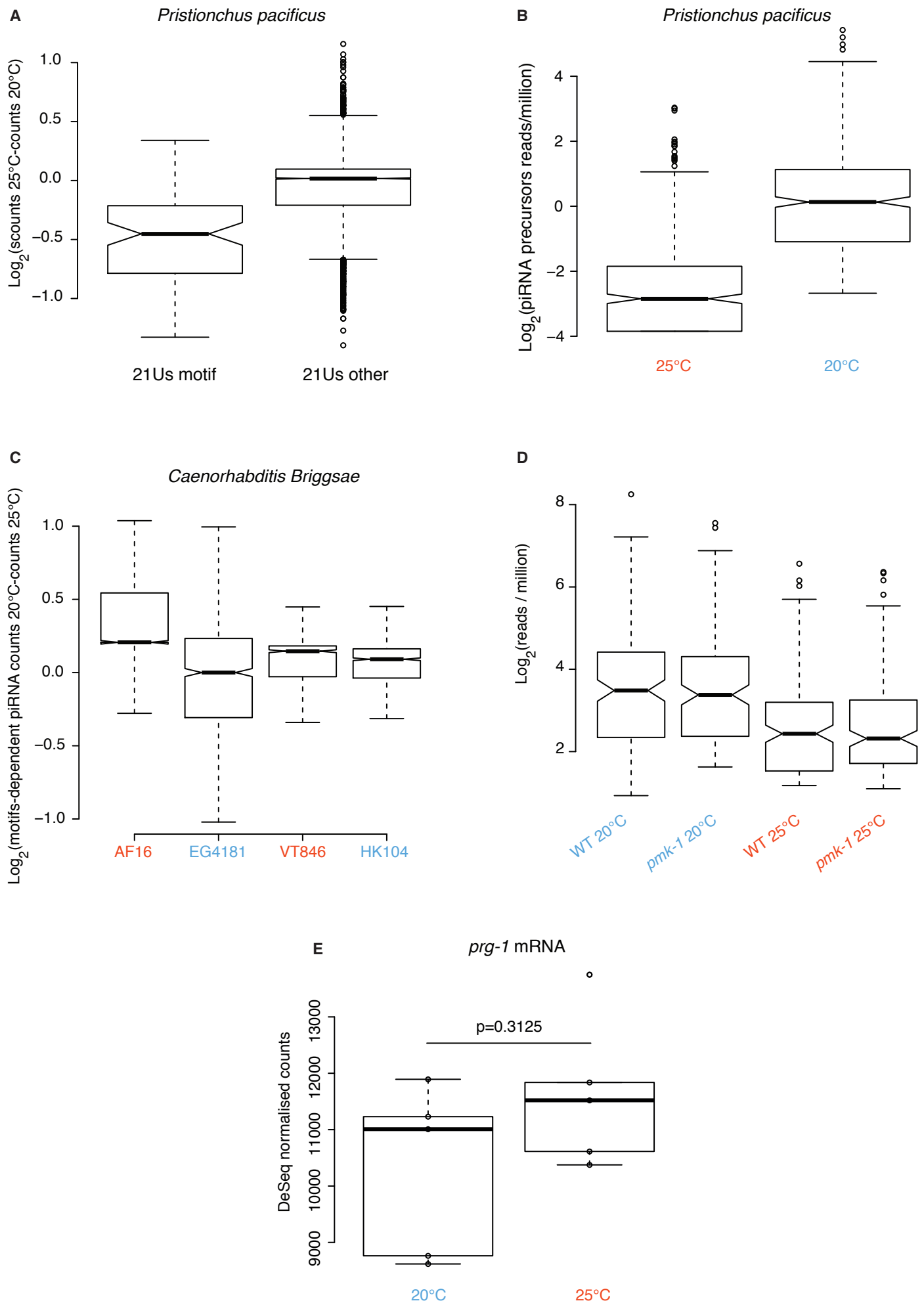

Figure S2

Supplement: Supplementary file 2 — Figure S2. piRNA production in different species and in the pmk-1 mutant. (A) Log2 ratio of the count of 21U small RNA in P. pacificus between 25 °C and 20 °C. piRNAs, identified by the presence of a conserved upstream motif, are reduced relative to all other 21U-RNAs. 4956 such loci were detected and used for analysis. (B) Difference in motif-dependent piRNA precursors at 25 °C and 20 °C in P. pacificus. (C) Ratio of the amount of motif-dependent piRNAs at 20 °C over 25 °C in 4 different strains of C. briggsae. AF16 is the standard laboratory strain. In red are tropical strains and in blue are temperate strains. 2431 such piRNA loci were detected in the reference strain AF16 and used for analysis. (D) Log2 reads per million of motif-dependent piRNAs in the wild-type (WT) strain and in the pmk-1 mutant at 20 °C and 25 °C. (E) Change in prg-1 mRNA level between 20 °C and 25 °C. (PDF 536 kb) [file 12915_2018_571_MOESM2_ESM.pdf]

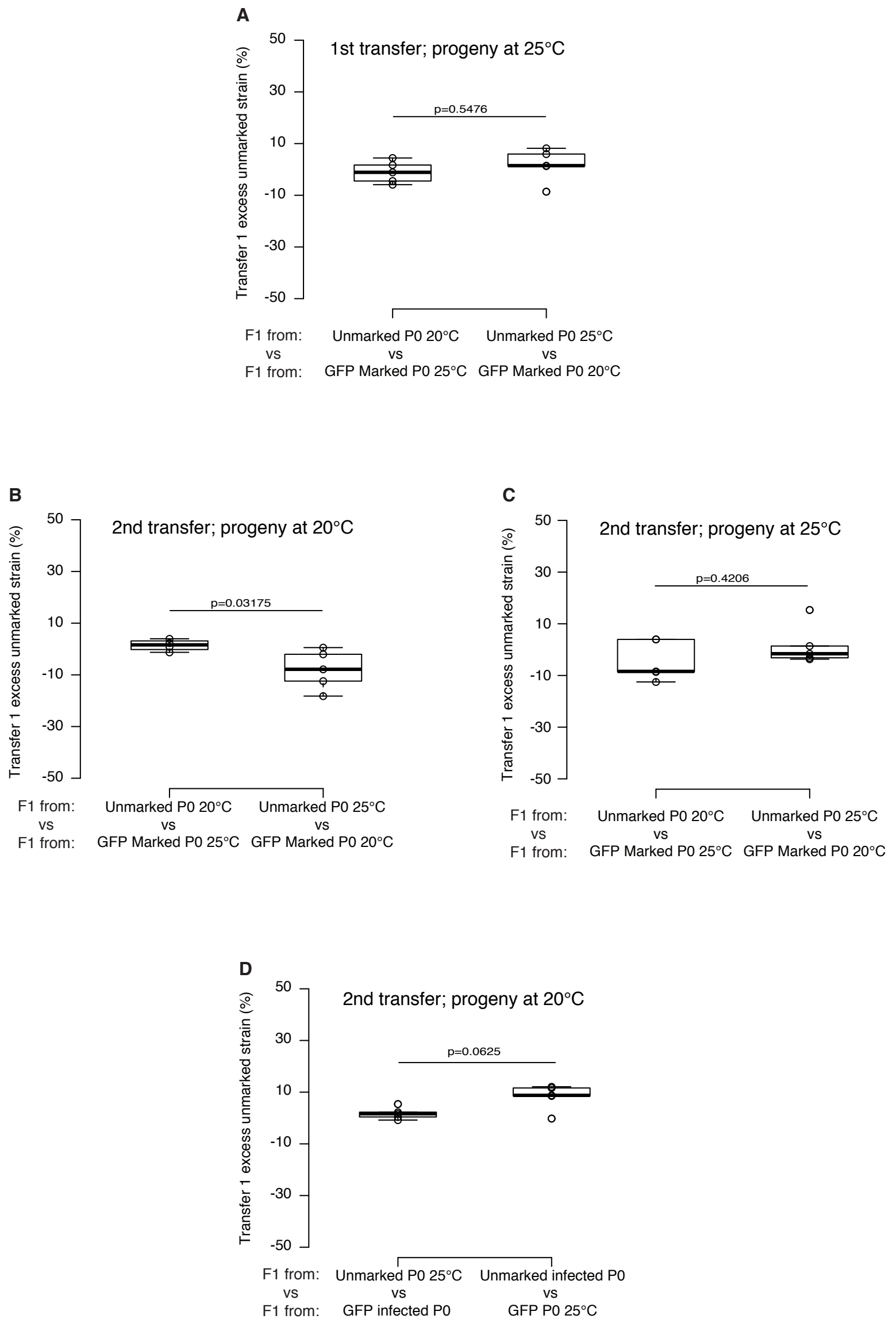

Figure S4

Supplement: Supplementary file 4 — Figure S3. Transgenerational alterations in gene expression induced by temperature. (A) Overlap between the change in gene expression at 25 °C compared to 20 °C in P0 (experiment 1) and the change in gene expression at 25 °C compared to 20 °C during the time course experiment (experiment 2). (B) Overlap between temperature-sensitive genes (grey) identified in (A), prde-1-dependent genes (blue) and prg-1-dependent genes (pink). (C) Overlap between temperature-sensitive genes in parents (grey), altered genes in prde-1 mutants (blue) and altered genes in F1 grown at 20 °C from parents grown at 25 °C (orange). (PDF 409 kb) [file 12915_2018_571_MOESM4_ESM.pdf]

**A**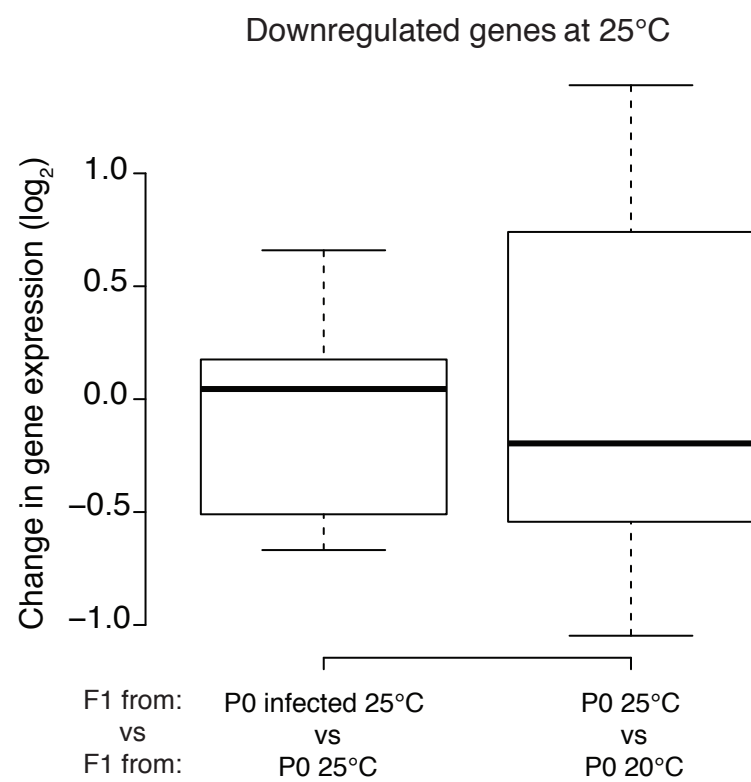**B**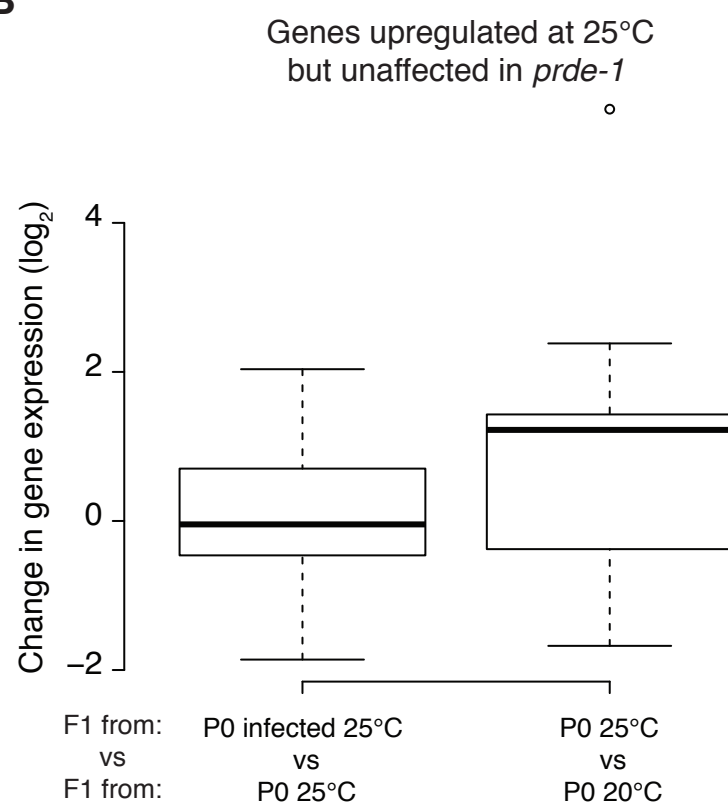

Figure S5

Supplement: Supplementary file 5 — Table S2. Proportions of the different strains from the competition experiments. (PDF 392 kb) [file 12915_2018_571_MOESM5_ESM.pdf]
