# Supplementary material for: The piRNA pathway responds to environmental signals to establish intergenerational adaptation to stress
Source: BMC Biol. 2018 Sep 18;16:103. doi: 10.1186/s12915-018-0571-y (PMC6145337; doi:10.1186/s12915-018-0571-y)
Supplement: Supplementary file 3 — Table S1. Genes identified as consistently changed at 25 °C compared to 20 °C. (PDF 368 kb) [file 12915_2018_571_MOESM3_ESM.pdf]

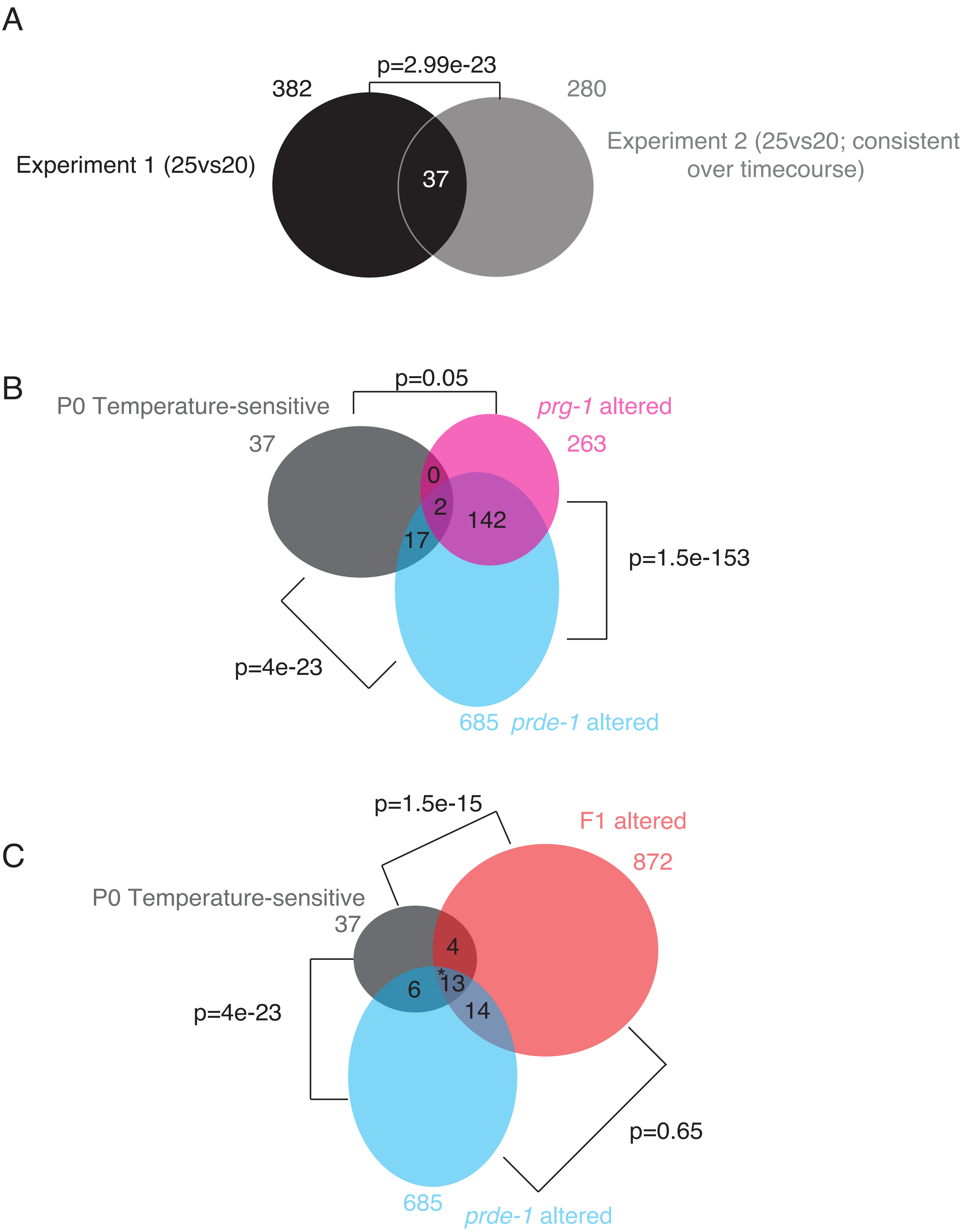

*p=0.008 (*prde-1* regulated genes are more likely to be transmitted intergenerationally than expected)

Figure S3
